# Effective Polarizability in Near-Field Microscopy of Phonon-Polariton Resonances

**DOI:** 10.3390/nano15060458

**Published:** 2025-03-18

**Authors:** Viktoriia E. Babicheva

**Affiliations:** Department of Electrical and Computer Engineering, University of New Mexico, MSC01 1100, 1 University of New Mexico, Albuquerque, NM 87131, USA; vbb@unm.edu

**Keywords:** optical resonances, van der Waals, hexagonal boron nitride, hyperbolic dispersion, phonon polaritons, polar dielectric, silicon carbide, nanoscopy, hot spots, polarizability

## Abstract

We investigate the resonant characteristics of planar surfaces and distinct edges of structures with the excitation of phonon-polaritons. We analyze two materials supporting phonon-polariton excitations in the mid-infrared spectrum: silicon carbide, characterized by an almost isotropic dielectric constant, and hexagonal boron nitride, notable for its pronounced anisotropy in a spectral region exhibiting hyperbolic dispersion. We formulate a theoretical framework that accurately captures the excitations of the structure involving phonon-polaritons, predicts the response in scattering-type near-field optical microscopy, and is effective for complex resonant geometries where the locations of hot spots are uncertain. We account for the tapping motion of the probe, perform analysis for different heights of the probe, and demodulate the signal using a fast Fourier transform. Using this Fourier demodulation analysis, we show that light enhancement across the entire apex is the most accurate characteristic for describing the response of all resonant excitations and hot spots. We demonstrate that computing the demodulation orders of light enhancement in the microscope probe accurately predicts its imaging.

## 1. Introduction

Photonic materials supporting phonon-polariton excitations with low losses in the mid-infrared (mid-IR) range have garnered significant attention recently [1,2,3,4,5,6,7,8,9,10,11,12,13,14,15]. The nonradiative optical loss in the structures made of such materials with phonon-polaritons is considerably lower compared to the plasmonic resonant response of metallic structures. In particular, silicon carbide (SiC) exhibits metal-like properties and has a negative permittivity in the spectral range near 11 µm [6,7,8,9]. It is related to SiC being a polar dielectric material characterized by its strong ionic bonding and ability to support optical phonon modes. Materials with phonon-polaritons exhibit strong light–matter interactions within their Reststrahlen bands, enabling sub-diffraction confinement and anisotropic propagation of infrared waves. While isotropic materials display metal-like properties in this regime and can support strong resonances, anisotropic materials are particularly intriguing due to their directional optical responses and hyperbolic dispersion. Anisotropic materials such as hexagonal boron nitride (hBN), alpha-phase molybdenum trioxide (α-MoO_3_), and gallium oxide (Ga_2_O_3_) demonstrate exotic polaritonic behaviors, including hyperbolic dispersion and directional control of energy flow. Other van der Waals materials, such as transition metal dichalcogenides, also exhibit strong optical anisotropy due to their layered crystal structure, leading to polarization-dependent excitonic resonances, tunable birefringence, and directional light–matter interactions [16] that give rise to unique near-field and polaritonic phenomena.

Hexagonal boron nitride is a van der Waals material composed of layered structures held together by weak interlayer forces, enabling unique optical and electronic properties. Because hBN is a layered material, it is actively studied due to its low optical losses and intrinsic hyperbolicity, which is a rare natural property [2,14,15,17,18]. Similar to artificial structures [17,19,20,21], hBN exhibits pronounced material anisotropy, with tensor terms of anisotropic permittivity becoming negative in specific spectral regions, known as Reststrahlen bands: around the wavelength of 7.3 µm (the wavenumber of 1370 cm^−1^, upper band) and 12.8 µm (780 cm^−1^, lower band). The spectral region λ ≈ 6.2–7.3 µm is notable for hBN exhibiting hyperbolic dispersion [19]. The so-called Type II hyperbolicity of isofrequency contours in the three-dimensional space of the wavevector occurs when a material’s permittivity tensor components have opposite signs, with one (typically out-of-plane) being positive and the other two (typically in-plane, negative) leading to hyperbolicity that allows extreme anisotropic light propagation. Unlike complex designs that require alternating metal–dielectric layers [20] or nanowire arrays [19], hBN layers can efficiently confine light and allow the design of optical elements and photonic components, such as antennas [22,23,24,25], hyper-lenses [26,27], waveguides [28], and tapered slabs [29].

Scattering-type scanning near-field optical microscopy (s-SNOM) can achieve near-field imaging far beyond the diffraction limit and unprecedented spatial resolution and is widely used for the optical characterization of material surfaces [30,31,32,33,34,35,36,37,38,39,40]. It enables analysis of sample permittivity [41,42,43], phase separation and differentiation of metallic and insulating particles [44], surface modes [45,46,47], and more. A key application of s-SNOM is investigating the resonant response of the nanostructure in the particular spectral range, including plasmon- and phonon-polaritons. High-quality-factor resonant excitations in structures can lead to significant variations in the s-SNOM response [48], with features in the imaging strongly influenced primarily by the scattering of the probe and other components of the system. Measurements obtained with s-SNOM reliably capture subwavelength features of structures and near-field increases in specific regions, including hot spots, edges, and nanoantenna resonance. However, the theoretical prediction remains constrained by the dipolar approximation of probe scattering [49,50,51,52,53]. Efforts to quantitatively describe s-SNOM results and correlate them with the near-field properties employing novel approaches are ongoing [54,55,56,57,58,59,60,61,62,63], including a recent method for reconstructing vertical dipolar interactions [63]. Recent work has reported good agreement with experimental measurements when the s-SNOM signal is estimated through the near-field signal by integrating the vertical component of the electric field over the surface of the tip [58]. As outer bright fringes and low-contrast lines have been observed in experiments [64], we first used Fourier demodulation analysis in combination with full-wave numerical simulations of a probe–nanostructure system in [43], which has further been expanded by other groups [56,57,58,59].

Here, we advance the theoretical modeling of the s-SNOM measurements by examining the connection of near-field increases in the probe–nanostructure system and s-SNOM characterizations. We investigate s-SNOM responses in structures supporting phonon-polariton resonances: structures with well-established resonance conditions (such as flat surfaces and nanospheres with metal-like properties), the edge of SiC with phonon-polariton resonant excitations (treated as an isotropic permittivity tensor with negative components), and the boundary of hyperbolic materials, namely hBN, featuring multiple reflections at the edges and highly localized phonon-polariton beams. We analyze edges that serve as examples of configuration where, in resonance conditions, the s-SNOM signal exhibits additional peaks and the outer bright fringes are related to the strongest resonant excitation. To surpass the dipolar model, we calculate demodulation orders of field enhancement in the probe, providing a more comprehensive account of hot spots and scattering variations. We establish a link between the probe’s effective polarizability and field enhancement in various regions of the nanostructure. We observe that reflection dominates and the far-field background is strong when the system is outside resonance conditions. In this case, the effective polarizability of the probe increases, enhancing the fields. Conversely, in resonant structures, field enhancement is associated with a reduction in the effective polarizability. We show the applicability of our technique to complex geometries with pronounced resonances, even when the dipolar approximation for the probe fails to hold.

## 2. s-SNOM Signal Harmonics

We develop a framework that includes rigorous wave-based computations of probe–nanostructure near-field interactions with Fourier demodulation analysis of the signal at higher orders in accordance with practical s-SNOM measurements. Typically, s-SNOM measurements are conducted in tapping mode; that is, the probe oscillates with a frequency *f*_T_ of approximately a couple hundred kilohertz. In this case, the probe oscillates above the sample with an amplitude in the order of tens of nanometers (we use the 60 nm amplitude). The near-field signal is modulated by this tapping because the probe–sample interaction is a function of the distance from the sample. Subsequently, the near-field signal is demodulated at harmonics of the tapping frequency (2*f*_T_, 3*f*_T_, etc.), and thus, the far-field background and scattering from the probe shaft and other parts of the probe can be suppressed. Our numerical simulations employ the same demodulation procedure for direct comparison with s-SNOM measurements. We accomplish this by conducting simulations at various probe–sample separations with uneven steps to account for fast changes near the sample. The probe–sample separation is the same as the probe heights above the surface for the flat part of the sample. For each static position of the probe, we determine the electromagnetic field distribution used in the calculations of reflectance and effective polarizability. To account for the tapping motion, we repeat the simulation for various probe heights above the sample. We then demodulate the signal using a fast Fourier transform. This mimics an approach curve replacing changes in time with changes at different vertical positions. This produces computed harmonics whose strength and phase angle can be directly matched with the near-field intensity and phase measurements.

We conduct comprehensive electromagnetic modeling of the nanostructure with practical geometry and material optical response using the CST Studio Suite package. The nanostructures under consideration are positioned on a Si substrate. The probe apex is spherical with a radius of 30 nm, and the entire probe shape is a cone narrowed at the tip with a radius of 300 nm and a height of 1 µm. Numerical modeling with varying distances and a minimum probe height of 1 µm shows the consistency of the simulation results. To accurately resolve subwavelength features without excessive computational overhead, ensuring simulation convergence requires adaptive meshing near regions of high field enhancement, particularly at material interfaces and probe apex. The boundary conditions are set to perfectly matched layers to minimize artificial reflections at the simulation domain edges, while periodic boundary conditions are employed in the lateral direction to model extended structures. Convergence tests are performed by refining the mesh and monitoring variations in near-field intensity and effective polarizability until changes remain below 1 × 10^−3^ across successive iterations, ensuring reliable numerical stability.

The probe’s bulk material is Si, chosen for its excellent mechanical properties and its ability to achieve an exceptionally sharp tip due to its crystalline planes, ensuring high-resolution performance. Si’s well-defined crystalline structure allows for the fabrication of sharp tips through anisotropic etching, where the slower etch rate of {111} planes creates precise, high-aspect-ratio geometries. This sharpness is critical to achieving detailed resolution in nanoscale measurements. A 2 nm Pt coating enhances the probe’s functionality by improving its durability and providing a robust surface layer. This Si-Pt combination is a standard configuration in atomic force microscopy, offering a balance of sharpness, stability, and reliable material characteristics, and has been adopted in s-SNOM setups. At a wavelength of 10.55 µm, its permittivity ε_Pt_ = −1441 + 1040i (data from [65]), and it closely resembles the behavior of a perfect electric conductor (PEC) with effectively infinite conductivity. The reflection from the 2 nm Pt is 97%, and the transmittance through a freestanding sample is only 0.34%. Thus, in the mid-IR range, the 2 nm Pt coating behaves similarly to a PEC, shielding the optical signal from propagating into the Si bulk, and thus, the probe’s Si material has no significant influence on the measurement.

**Figure 1:**
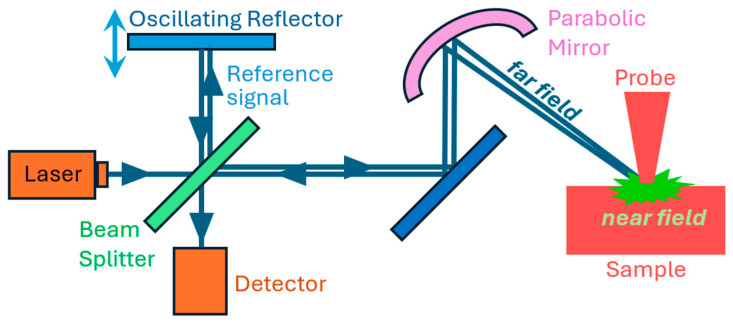
Schematic illustration of the experimental s-SNOM setup. Instead of a conventional heterodyne approach where the reference beam is frequency-shifted, pseudoheterodyne detection modulates the reference beam’s phase at a certain frequency, often via a vibrating reference mirror.

The effective probe–nanostructure polarizability is $\alpha (x,d)=p_{1}p_{2}r(x,d)/(2\pi \beta \tan\psi )\cdot \exp[-2i\beta (z_{\mathrm{top}}-z)\cos\psi ]$, where *r*(*x*,*d*) is the reflectance coefficient acquired from the full-wave simulation and is a function of the probe distance to the nanostructure, *p*_1_ and *p*_2_ are the size of the simulation region in the *x*- and *y*-directions, respectively, β is the wavevector of the impinging light, ψ = 45° is the angle of the beam, *z*_top_ stands for the top edge of the modeling region, and *z* stands for the vertical position of the nanostructure or the flat interface. The effective probe–nanostructure polarizability α(*x*,*d*) is utilized to determine the field scattered in the far-zone $E_{\mathrm{scat}}(d)=E_{\mathrm{scat}}(d_{0}+\Delta d\sin(\omega_{T}t))=\sum_{n}E_{n}e^{in\omega_{T}t}$, where *E_n_* denotes the complex field in the *n*-th demodulation harmonic, *t* is time, *f*_T_ ≈ 0.3–0.03 MHz corresponds to the probe frequency, and ω_T_ = *f*_T_/2π. Additionally, far-field radiation *E*_scat_ is mixed with a reference signal *E*_R_ sent back from an oscillating reflector with *f*_R_ ≈ 0.3 kHz, mimicking a pseudoheterodyne interferometer setup (Figure 1). Lastly, the amplitude of the signal is numerically demodulated and normalized by dividing the response at a specific coordinate point of the nanostructure by the response at the bare substrate or flat interface located far from the nano features, that is, $P_{n}=\left|E_{n,\;\mathrm{nano}}\right|/\left|E_{n,\;\mathrm{sub}}\right|.$

The modeling is conducted for the entire geometry with the probe located at varying distances (with a minimum of 2 nm above the nanostructure), considering a Δ*d* = 60 nm amplitude of probe oscillations. The points are distributed irregularly to adjust for the fact that most variations in effective polarizability take place within the initial 10s of nanometers from the nanostructure. Thus, in contrast to earlier techniques, our approach incorporates realistic probe and sample structures, though the calculations are restricted by the dipolar assumption of probe-scattered waves. We link the near-field enhancement to variations in the effective polarizability of the probe accounting for the nanostructure presence. The tapping mode of s-SNOM is simulated by performing full-wave electromagnetic calculations at multiple discrete probe heights above the sample, capturing the variations in near-field interactions as the probe oscillates. The resulting near-field signal is then demodulated using a fast Fourier transform to extract higher-order harmonics, mimicking the experimental detection process and suppressing background contributions.

Numerical simulations are conducted in p-polarization for the nanostructures with practical dimensions, geometries, material optical properties, substrates, and other layers. We examine the following characteristics derived from the computations and their dependence on the vertical position of the probe (defined as *z*_probe_ = *d*_0_ − Δ*d*, so the minimum value is 2 nm) as well as the lateral position of the probe *x*_probe_. The analysis includes (1) polarizability of the probe |α|, calculated based on the reflectance of the probe–nanostructure system for dipolar scattering; (2) s-SNOM response derived based on the probe’s polarizability; (3) field enhancement derived from the nonradiative loss of energy, that is, absorption and heat dissipation in the probe apex (bottom hemisphere) as well as Fourier demodulation harmonics of this characteristic, specifically second, third, and fourth orders; and (4) integral near-field increase and hot spots in various regions of the nanostructure.

The probe’s effective polarizability is derived from the reflectance of the system, and the s-SNOM response is subsequently computed. Whenever the effective polarizability is analyzed, we plot the absolute value of this complex quantity, |α|. This approach also allows us to determine additional quantities of interest, including the optical field at specific regions of the probe or nanostructure and, more broadly, localized energy dissipation within regions of the nanostructure. This information is particularly valuable for gaining an overall understanding of the field distribution in cases where the locations and number of hot spots, such as the multiple hot spots associated with edge characterization, are unknown. This work presents a numerical study of the s-SNOM response arising from the resonant response of nanostructures with phonon-polaritons, extending beyond the simple dipolar approximation. Due to the nanoscale nature of the tip, higher-order dipole contributions are also expected to influence the response. The study investigates how the tip’s position affects light–matter interactions and examines various phonon-polaritonic materials, such as isotropic SiC and hyperbolic hBN. These finite-size phonon-polariton structures are garnering significant attention in the fields of photonics and 2D materials. The key difference between the SiC and hBN cases lies in the presence of edge phonon-polaritonic modes: SiC supports these modes, while hBN does not (excluding the propagating mode along the edge). This work presents numerical calculations exploring unique electromagnetic features observed in s-SNOM maps of SiC and hBN thin films with sharp edges under optical excitation that are either resonant or non-resonant with the material’s phonon-polaritons. The theoretical framework provides a systematic explanation of the complex electromagnetic phenomena, and the distinction between resonant and non-resonant responses provides insight into their respective resonant and non-resonant behaviors.

The cumulative field localization and enhancement *W* is proportional to ~*E*^2^, which can conveniently be calculated as the power loss given directly by the CST Studio Suite. The intensity of resonant excitations in the system can be associated with absorptance, serving as a practical measure. The relationship can be understood by examining the rate of dissipated energy within the system and connecting it to the overall enhancement of the electromagnetic field. The rate of dissipated energy can be calculated as

$$W={∭}_{V}\sigma {E(x,y,z)}^{2}dxdydz=\approx \frac{1}{2}{\omega \varepsilon }^{\prime \prime }{∭}_{V}{\left|E(x,y,z)\right|}^{2}dxdydz,$$

where *V* corresponds to the system volume where the dissipation is analyzed, ω denotes the oscillation frequency of the incident wave with the electromagnetic field, σ is conductivity, ε″ is the permittivity’s imaginary component, and ***E***(*x*,*y*,*z*) stands for the electric field. The intensity and spectral location of the resonant excitations serve as indicators of enhanced electromagnetic fields and energy confinement, thus linking absorptance to field enhancement and overall resonant mode localization.

Any variations in *W* magnitude and integral over the probe tip reflect contributions from multiple hot spots within the nanostructure. Analogous to calculating the s-SNOM response using polarizability α at various vertical coordinates *z*_probe_, we determine the Fourier demodulation harmonics of field enhancement from computations at various *z*_probe_ values. Consequently, the primary benefit of this method lies in its ability to go beyond the dipolar approximation and reflectance-based calculations, capturing all field enhancement spots in the integrated probe–nanostructure system. In what follows, we present a comparison between results derived from effective polarizability and field enhancement.

## 3. Resonant Response of the Interface and Sphere

**Figure 2:**
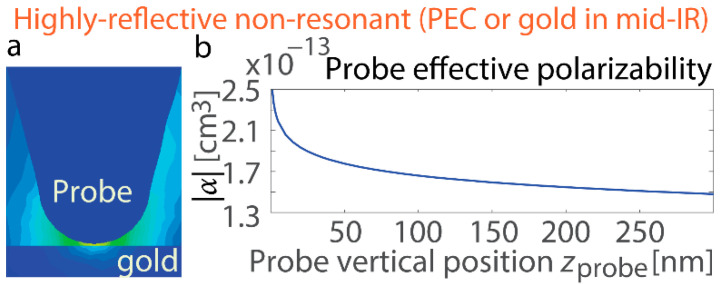
Non-resonant interface. (**a**) Schematic of the s-SNOM probe on top of a highly reflective non-resonant interface (Au in the mid-IR spectrum). The lighter color on the map indicates higher electric field intensity, and the dark blue color corresponds to a near-zero electric field. (**b**) Variation in the probe’s dipolar polarizability as it moves away from the gold interface. The steady decline agrees with typical approach-curve observations.

To start with, we analyze the simplest case that either exhibits or lacks resonance. The flat surface serves as a reference system, allowing the characterization of phonon-polariton resonances in a well-defined geometry before extending the analysis to more complex edge structures. By first studying the near-field interactions on a planar interface, the work establishes a baseline for understanding resonance conditions and field enhancements, which are later compared to the more intricate behavior observed at material boundaries. A common non-resonant case is a highly reflective interface, like Au in the mid-IR region. At a wavelength of 10.55 µm, its permittivity ε_Au_ = −5100 + 970i (data from [66]), and it largely mimics the behavior of PEC with virtually infinite conductivity. Simulations reveal that the polarizability of the probe placed above such a non-resonant surface decreases monotonically with increasing vertical position *z*_probe_ (Figure 2). A similar consistent drop is detected in other parameters, including field enhancement in the probe and field enhancement at the probe [43], ultimately leading to the same trend in the s-SNOM response observed in experimental approach curves. The approach curve in the s-SNOM measurements refers to the variation in the scattered signal as the probe tip approaches the sample surface. It provides critical information about the near-field interaction strength and serves as a basis for separating near-field and far-field contributions.

**Figure 3:**
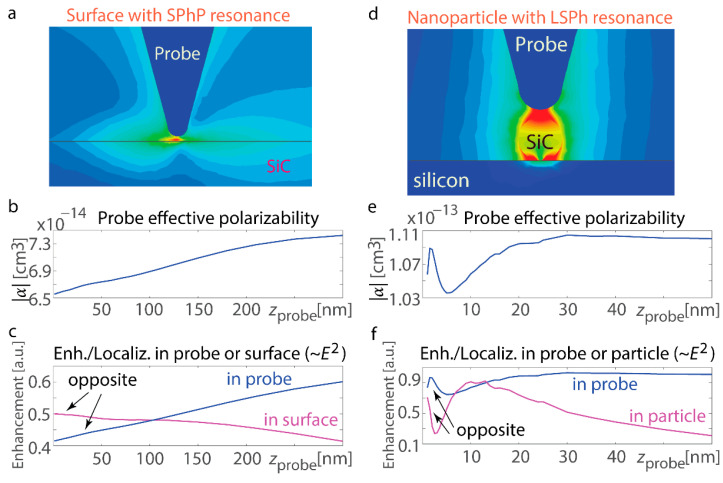
Resonant structures. (**a**) Probe and electromagnetic profiles for the scenario of an interface that supports SPhP resonant excitation (flat SiC boundary with ε_SiC_ = −1 + 0.08i in free space). The dark red color on the map indicates higher electric field intensity, and the dark or light blue color corresponds to a near-zero electric field. (**b**) Polarizability |α| of the probe rising on top of the resonant interface. It experiences steady enhancement for *z*_probe_ < 300 nm. (**c**) Variation in field enhancement and localization in the probe and the interface supporting SPhP resonances (0.2 × 0.2 × 0.2 µm^3^ region). The field enhancement on the interface surface exhibits an *inverse* trend compared to that in the probe and its polarizability. (**d**) Probe and field enhancement for the scenario of the nanosphere with LSPh resonance (ε_SiC_ = −2 + 0.11i, corresponding to the resonance in the nanosphere; refer to the equation in the text). The nanosphere is placed on a silicon substrate. The dark red color on the map indicates higher electric field intensity, and the dark or light blue color corresponds to a near-zero electric field. (**e**) Effective dipolar polarizability of the probe rising vertically on top of the resonant nanosphere: the irregular trend for *z*_probe_ < 30 nm transitioning to a reduction at greater separations. (**f**) Variation in field enhancement and localization in the apex of the probe and the resonant nanosphere: Field enhancement in the nanosphere exhibits an inverse trend compared to that in the probe and its effective dipolar polarizability.

We conduct computation for the interfaces that exhibit resonant responses and demonstrate that their features differ significantly (Figure 3). If ω stands for the angular frequency and *c* for the speed of light in vacuum, an interface with negative permittivity $\varepsilon_{\mathrm{SiC}}$ can support the propagating surface mode with a wavenumber $k_{\mathrm{SPhP}}=(\omega /c)\sqrt{\varepsilon_{\mathrm{SiC}}/(\varepsilon_{\mathrm{SiC}}+1)}$ and therefore exhibits resonance when Re[ε_SIC_] = −1. For this study, we consider a SiC surface at a wavelength of 10.6 µm where its ε_SiC_ = −1 + 0.08i. Notably, this is not merely a condition for exciting and propagating a surface phonon-polariton (SPhP). Instead, the singularity represents an SPhP resonance due to the near-zero divisor in the wavenumber *k*_SPhP_. These SPhP resonant excitations result in a strong but finite rise in optical field intensity due to the dumping introduced by the non-zero Im[ε_SIC_] component (Figure 3b,c). For *z*_probe_ < 300 nm, both the probe’s polarizability and the near-field increase within it show trends opposite to those observed for the non-resonant surface: both increase monotonically. For *z*_probe_ > 300 nm, the effective polarizability drops, resembling the behavior seen with non-resonant interfaces (beyond the extent of the plot). By comparison, field enhancement near the resonant interface peaks when the probe is in close proximity. These results confirm that resonance excitation on the surface significantly suppresses scattering from the probe, leading to a reduction in the effective polarizability.

This observation is further supported by computations for a resonant nanosphere (*R* = 30 nm) exhibiting a localized surface phonon (LSPh) resonance (Figure 3d). The analysis of the nanosphere provides insight into localized surface phonon resonances, offering a complementary perspective to the extended modes observed on flat surfaces. By studying a finite structure with strong field confinement, the nanosphere case helps distinguish the effects of geometry on near-field enhancements and resonance shifts, which are crucial for understanding edge and boundary interactions in more complex nanophotonic systems. In the quasi-static approximation, the field enhancement within the sphere is proportional to $\varepsilon_{\mathrm{SiC}}/(\varepsilon_{\mathrm{SiC}}+2)$, and resonance occurs at Re[ε_SiC_] = −2. To leverage the standard mathematical construct, we consider a spherical nanoparticle with permittivity ε_SiC_ = −2 + 0.11i, corresponding to λ = 10.7 µm. A key advancement here is the calculation for a sphere exhibiting localized surface phonon resonance. Unlike previous studies focusing on semi-infinite sharp edges, this study considers finite-size structures with localized surface polaritons. These sharp-edged, finite-size structures, made from materials with resonant responses and hyperbolic dispersion, hold promise for future applications.

Numerical simulations reveal non-monotonic variations in the probe’s effective polarizability and field enhancement, along with opposing trends in field enhancement within the probe and the nanosphere (Figure 3e,f). From the field distribution and full-wave computations, we further analyze the probe’s polarizability as the probe rises on top of the in-resonance nanosphere. There is also an irregular trend for *z*_probe_ < 30 nm, as well as a decline at greater distances. In Figure 3f, the opposing trends in enhancement between the probe and the particle are observed at various *z*_probe_ probe positions (labeled “opposite” in the figure). Rapid variations occur at small distances, likely undetectable in experiments, but similar behavior is evident at larger *z*_probe_ values, where both quantities exhibit monotonic changes.

The absorption trends differ, with the sphere and tip showing opposite tendencies in both absorption and effective polarizability. These changes occur over shorter distances between the probe and the surface in the case of the nanosphere as opposed to the flat surface. This difference arises because the field enhancement is highly localized around the nanosphere, whereas it extends over a greater distance for the flat surface—a distinction evident when comparing their respective field distributions (see Figure 3a,d). Furthermore, the relative variation in the polarizability with distance, shown in Figure 3e for the finite-size particle, is significantly smaller than that in Figure 3b for the surface. This difference can be attributed to the nanoscale dimensions of the sphere, which result in a comparatively smaller impact than that of an infinitely extended surface.

This advanced theoretical model improves upon conventional approaches by incorporating the probe’s effective polarizability while establishing a more direct connection between near-field enhancements and s-SNOM characterizations. Unlike previous models that primarily rely on simplified scattering assumptions, it explicitly accounts for the spatially varying probe–nanostructure interactions across different resonant geometries. By performing demodulation analysis of the near-field signal, the model provides a systematic way to interpret s-SNOM measurements in configurations where hot spots and field distributions are not trivially predictable. This enables a more reliable theoretical framework for analyzing resonant excitations in phonon-polaritonic and other nanoscale material systems.

The earliest research on s-SNOM has shown that the lateral resolution of s-SNOM is primarily limited by the tip’s radius of curvature, which is typically between 10 and 20 nm, and is mainly independent of the probing light’s wavelength [67,68]. It has also been demonstrated that for a metal probe with an extremely sharp apex, the s-SNOM signal diminishes over a 5 nm length scale [69]. For a flat interface between two materials, employing tips with a typical apex diameter of approximately 46 nm has revealed that the material boundary in s-SNOM images appears around 20 nm wide [70]. Additionally, the near field at the tip apex is significantly screened on the highly reflective (metallic) side, diminishing the apparent boundary width in s-SNOM images. Furthermore, tip radii ranging from 10 nm to 200 nm can lead to notable differences in the observed near-field contrast and the demodulated signal strength [71]. Although increasing the tip radius from 10 nm to 200 nm (a 20-fold increase) does not proportionally degrade the resolution, the resolution is only modestly affected, indicating that factors other than tip radius also play significant roles in determining s-SNOM resolution.

**Figure 4:**
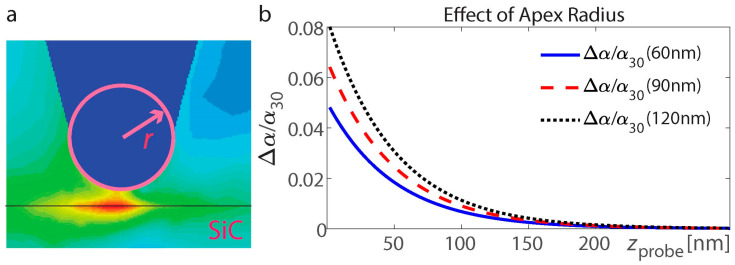
(**a**) Probe schematic and apex radius definition. The dark red color on the map indicates higher electric field intensity, and the dark or light blue color corresponds to a near-zero electric field. (**b**) Effect of the apex radius on the effective polarizability of the tip: Variation in the relative change in probe polarizability $(\alpha_{r}-\alpha_{30})/\alpha_{30}$ (for *r* = 60, 90, and 120 nm) for the probe radius deviating from the initial value *r* = 30 nm. The flat SiC interface supports SPhP resonant excitation for ε_SiC_ = −1 + 0.08i in free space.

The variations in probe geometry, such as changes in tip radius, can slightly affect the near-field interactions and the resulting s-SNOM signal. We analyze the effect of the apex radius on the effective polarizability of the tip by calculating the relative change in probe polarizability $(\alpha_{r}-\alpha_{30})/\alpha_{30}$ for the probe radius deviating from the initial value of 30 nm, that is, *r* = 60, 90, and 120 nm for the flat SiC interface supporting SPhP resonant excitation for ε_SiC_ = −1 + 0.08i in free space (Figure 4). The relative change in probe polarizability increases with an increase in the apex radius, which is consistent with the tendency for larger scatterers and increased area of interaction. Furthermore, a smaller tip radius enhances spatial resolution and increases sensitivity to localized field variations, but in practice, it also reduces the interaction volume, leading to weaker signal strength and increased sensitivity to noise. Conversely, a larger tip radius averages the near-field response over a broader region, which can obscure fine details but provides a more robust signal.

Material properties, such as doping levels in SiC, can also alter the near-field response: Higher doping concentrations introduce free carriers, increasing optical losses and shifting the phonon-polariton resonance, which can change the spatial localization of field enhancements and the effective signal offset from material edges. In heavily doped SiC, the increased carrier density screens phonon-polaritonic modes [72], reducing their confinement and modifying the demodulated near-field contrast. The sensitivity of the findings to these parameters suggests that practical comparisons must take into account probe and sample variations, as differences in tip geometry or material properties can lead to systematic shifts in observed resonance positions and near-field distributions.

## 4. Edge Resonances

Turning our attention to the edge, we now examine how the presence of a material boundary influences phonon-polaritonic resonances, alters near-field distributions, and introduces distinct scattering effects that are absent in flat surfaces and nanospheres. The analysis of the edge is essential for understanding how phonon-polaritonic resonances behave in confined geometries where reflection, scattering, and mode coupling play a significant role. By investigating near-field enhancements and signal demodulation at the boundary, this study reveals how edge-induced resonances differ from those on flat surfaces and nanospheres, providing critical insights for designing nanophotonic devices with tailored light confinement. When a distinct material boundary is approached, the s-SNOM response, particularly the probe’s effective dipolar moment and polarizability, undergoes notable changes. The edge measurements typically exhibit two characteristics: a wide decline (a subtle feature in the image map) that consistently emerges on the outer side of the layer boundary, regardless of the material properties, and a distinct increase (an outer prominent band in the image map) that varies depending on the reflection strength from the edge and its resonant excitations. The earlier developed methods assumed dipolar excitations in the probe and enabled the calculation of s-SNOM signals across various demodulation orders by simulating the entire structure, arbitrary probe positions, and vertical scans, and this approach is effective only for structures with a single hot spot, such as planar surfaces. However, an advanced model is needed for an accurate description of edge resonances. The broad signal decrease occurs due to the probe being effectively embedded in a reduced amount of optically dense material near the material boundary, reducing its polarizability and making its impact on near-field variations less significant. For an ideal right-angle boundary of a 60 nm thick material, this drop begins around *x*_probe_ = 50 nm, with the lowest signal occurring approximately at *x*_probe_ = −50 nm to −100 nm.

The sharp peak outside the edge can be used to classify edges into two types: non-resonant and resonant, depending on the edge geometry, material properties, and the efficiency of edge resonance excitation. For the boundary outside the resonance conditions, the signal increase on the outer part of the material boundary corresponds to the formation of two spots with field enhancement: one right beneath the probe and the second one between the probe and the vertical wall of the material boundary in the right-angle configuration (side hot spot [43]). For the boundary outside the resonance conditions, high demodulation harmonics of the s-SNOM response exhibit less prominent enhancement, frequently vanishing by the third or fourth order. The field scattered from the side hot spot varies more slowly than that from the hot spot beneath the probe on a flat surface. As the probe descends along the vertical wall of the material boundary, its position changes minimally affect the near-field response. Consequently, the s-SNOM response, which is sensitive to field enhancement, weakens at higher demodulation orders. This leads to a deeper, more pronounced broad dip in the signal, with the subtle feature appearing as a depression for the non-resonant boundary. The enhancement band on the outer part of the material boundary, influenced by both spots with field enhancement, diminishes with increasing demodulation order. These edges are categorized as non-resonant because the enhancement band inevitably coincides with the probe position consistent with the two-hot-spot excitation and weakens or vanishes at higher demodulation orders.

Contrary to the enhancement observed for non-resonant edges, the response peaks at the boundary that supports resonant excitations persist and become more pronounced at higher demodulation orders. As the probe moves along the vertical wall of the material boundary, it triggers the resonant response, with the excitation strength varying based on the probe position. Consequently, the signal maximum (fringe) occurs where edge resonance excitation is most efficient, which may not align with the two-hot-spot condition. The resonant excitations originate from various mechanisms, such as phonon- or plasmon-polariton, Fabry–Pérot bouncing from layer boundaries (e.g., hyperbolic rays in hyperbolic medium), and additional resonances that produce significant field enhancement near the edge. These resonances lead to sustained and intensified peaks at higher demodulation orders, distinguishing resonant edges from non-resonant ones.

The resolution of s-SNOM measurements surpasses the Rayleigh criterion and allows for resolving sub-diffraction details, with the s-SNOM resolution governed by the dimensions of the probe apex (~30 nm). Thus, the characteristic dimensions and the typical resolution are ~100 times smaller than the mid-IR wavelength. It is necessary to differentiate between edge features and patterns that emerge at the nanostructure interface due to the excitation of propagating surface and waveguide modes within one or several layers of the structure. Typically, to analyze mode dispersion in the nanostructure or plain layer, pattern examination involves a spatial Fourier transform (not to be confused with Fourier demodulation discussed here for vertical movement of the probe) [27,45]. In this situation, it is crucial to exclude the outer bright and dark bands from the decomposition. Furthermore, the pronounced outer bands provide insight into the optical properties of the nanostructures and materials involved. When dealing with different materials, for example, various nanoparticles or patches [44], the appearance of a bright band suggests the presence of particles with metallic properties.

**Figure 5:**
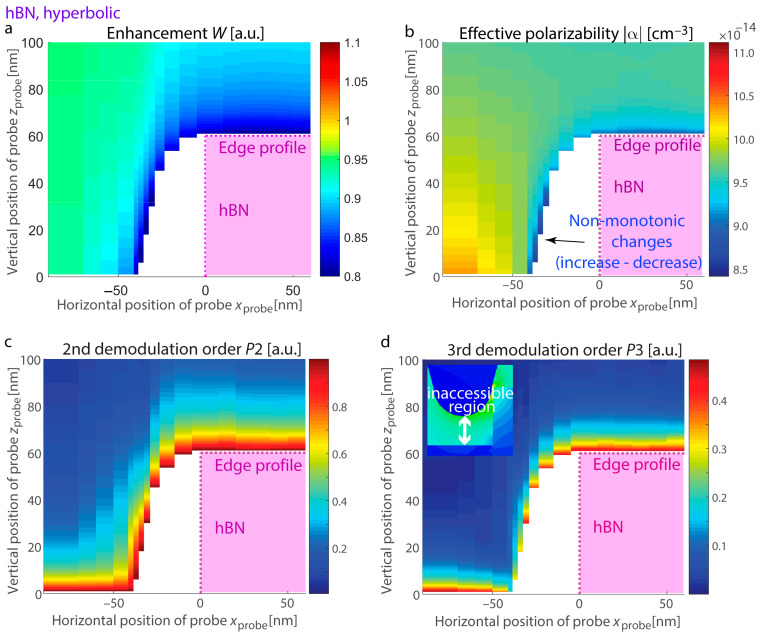
Vertical and lateral variations in the s-SNOM response at the boundary of hyperbolic hBN nanostructures (ε_hBN,||_ = −20 + 0.8i, ε_hBN,⊥_ = 2.8 + 8 × 10^−4^i, corresponding to 7.1 µm). (**a**) Enhancement *W*, (**b**) effective dipolar polarizability |α|, (**c**) 2nd harmonic demodulation of the s-SNOM response, and (**d**) 3rd harmonic demodulation of the s-SNOM response. Signals for (**c**,**d**) are derived by demodulating (**b**), representing a metric typically measured in s-SNOM experiments during either vertical (approach curves) or lateral mapping. Inset: Example of an inaccessible region due to the conical shape of the probe.

**Figure 6:**
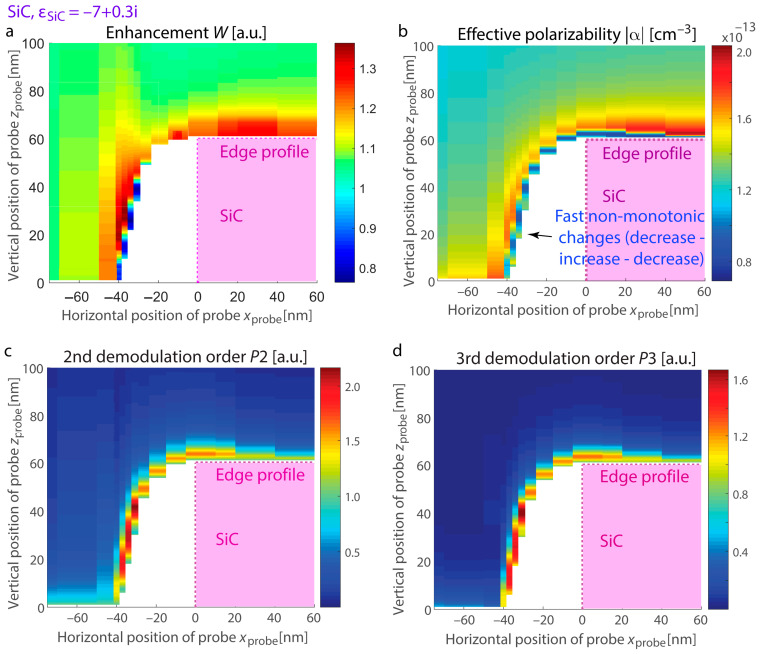
Vertical and lateral variations in the s-SNOM response at the boundary of in-resonance SiC nanostructures with ε_SiC_ = −7 + 0.3i. The quantities under consideration are the same as in Figure 5. (**a**) Enhancement *W*, (**b**) polarizability |α|, (**c**) 2nd harmonic of the s-SNOM response, and (**d**) 3rd harmonic of the s-SNOM response. The signals for (**c**,**d**) are derived by demodulating (**b**).

To predict the optical response at the boundary of thin layers supporting confined resonances, we examine hBN and SiC exhibiting distinct resonant excitations: hBN supporting hyperbolic dispersion with ε_hBN,||_ = −20 + 0.8i, ε_hBN,⊥_ = 2.8 + 8 × 10^−4^i at λ = 7.1 µm (permittivity is taken from [73], Figure 5) and SiC at a wavelength of λ = 11.3 μm with ε_SiC_ = −7 + 0.3i (Figure 6). In both systems, the material thickness is 60 nm, and the substrate is made of Si. We calculate the s-SNOM response with its vertical and horizontal variations as well as enhancement at the nanostructure edges with resonances. Due to the conical shape of the probe in s-SNOM, certain regions on the map become inaccessible as the probe cannot effectively interact with the sample at those points (inset in Figure 5d).

We observe that phonon-polariton resonant excitations in SiC are analogous to those in advanced nanostructures where the permittivities of adjacent materials have similar magnitudes but opposite signs for their real parts (for example, metal–dielectric systems in the visible spectrum). On the other hand, hBN exhibits resonances associated with the excitation and propagation of phonon-polariton rays within the layer. These rays propagate at specific angles determined by the permittivity tensor and undergo multiple reflections at the layer boundaries, as detailed in studies [21,26,27,74]. Fast variations and irregular trends for SiC in the response are evident as the probe rises vertically along the boundary. Due to the conical geometry of the probe, its trajectory deviates from the vertical contour of the edge, leading to white regions on the maps where the response value cannot be detected.

**Figure 7:**
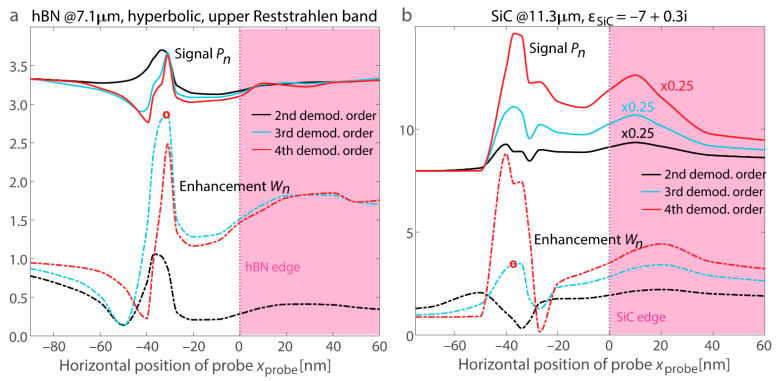
s-SNOM signal response *P_n_* and enhancement *W_n_* of the probe at the boundary of the in-resonance nanostructures. (**a**) Demodulation orders at the edge of the material exhibiting hyperbolic dispersion. (**b**) Harmonics for ε_SiC_ = −7 + 0.3i. In both panels, the response is rescaled to reach one at *x*_probe_ = −2 µm on the substrate for each demodulation order. For clarity, the data are vertically offset by 2.5 for hBN and by 7 for SiC. In both panels, solid and dot-dash lines of a particular color correspond to the demodulation order denoted in the legend. The boundary is visualized by a pink rectangle, and both cases are for a thickness of 60 nm. Round red marks denote the positions where Figure 8 is computed (*x*_probe_ = −31 nm for hBN and *x*_probe_ = −37 nm for SiC). For both materials, the matching behavior of the s-SNOM response *P_n_* and the harmonics of the field enhancement *W_n_* confirms that the resonance excitation locations are accurately represented by the models.

Figure 7 illustrates the scenario where the probe is positioned adjacent to the surface, showing a bright fringe at the edge for both materials. Due to the resonant nature of this fringe (as opposed to enhanced reflection from the spots with field enhancement), the increase persists in higher demodulation harmonics, and it appears more prominently. The calculated s-SNOM response and the demodulation components of field enhancement exhibit good agreement. Specifically, the peak positions align for both signal and field enhancement computations, and the maxima are consistently shifted from the location of the two-hot-spot configuration associated with higher reflectance. At the same time, for higher demodulation orders, the SiC s-SNOM response computed using the dipolar model shows a monotonous rise in the maximum prominence, likely an artifact of the dipolar approximation. Conversely, the demodulation orders of field enhancement exhibit only slight increases for higher harmonics, making them more suitable for describing phonon-polariton edge resonances.

The ~30–40 nm offset between the signal or absorption peak and the physical position of the edge along the *x*_probe_ axis is comparable to what is observed in experiments [43,64]. Edge sharpness plays a critical role, as sharper edges confine phonon-polaritons more tightly, reducing the offset, while rounded or less-defined edges distribute the field enhancement over a broader area, increasing the shift. A sharper transition can result in offsets of ~30–40 nm, whereas more gradual transitions may lead to higher displacements determined by the edge slope. A more detailed analysis incorporating these effects could further refine the interpretation of s-SNOM measurements and improve the accuracy of near-field mapping at material boundaries. The offset shows a slight dependence on the material’s optical properties but generally falls within several tens of nanometers. In practice, this distance is not only influenced by the sharpness of the edge but also by factors such as the degree of surface roughness and the precision of the edge definition. A sharper edge typically results in a more pronounced and localized signal, whereas a rounded or less-defined edge can cause the signal to spread out, further contributing to the observed offset.

**Figure 8:**
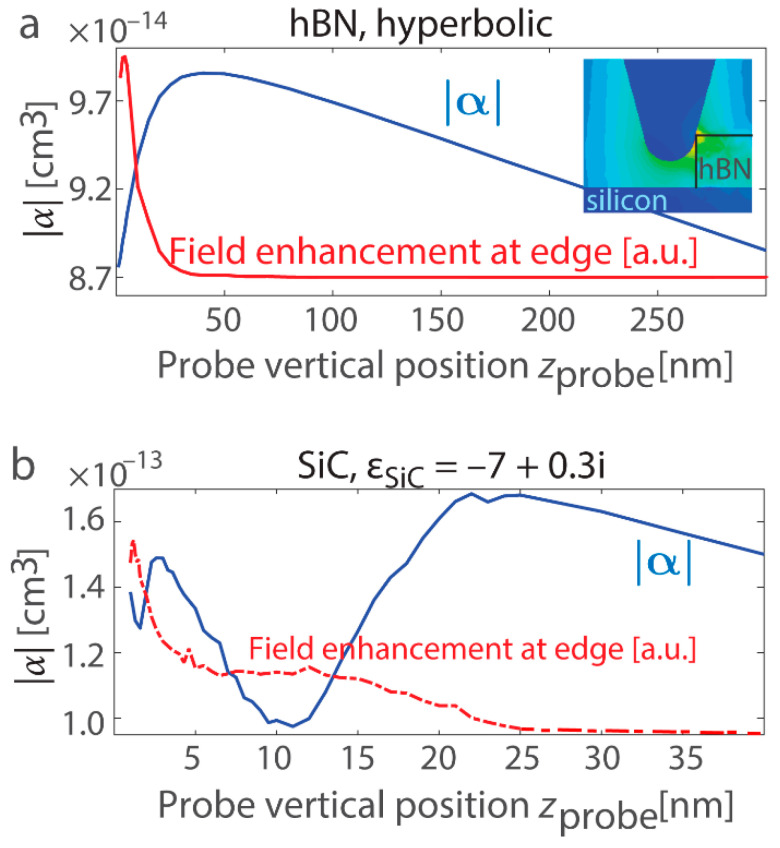
(**a**) Variation in the effective dipolar polarizability |α| generated in the probe at the hBN boundary as the probe approaches the interface. The effective polarizability decreases for *z*_probe_ < 20 nm, where the near field is significantly strengthened (an opposite trend like in Figure 3c,f). The plot corresponds to x_probe_ = −31 nm, aligning with the maxima of the 3rd and 4th demodulation harmonics. The maxima are shifted relative to the location where two spots with field enhancement are excited at once (probe/wall and probe/substrate at x_probe_ = −37 nm). Inset: Probe adjacent to the hBN layer when maximum edge resonance is excited with phonon-polariton ray generation. The yellow, green, and light blue colors on the map indicate higher electric field intensity, and the dark blue color corresponds to a near-zero electric field. (**b**) The same as (**a**), but for the SiC boundary. Within the first 25 nm of rising above the edge, there is a pronounced field increase and non-monotonic variations in the probe’s polarizability (once more, an opposite trend like in Figure 3c,f). The figure corresponds to *x*_probe_ = −37 nm, coinciding with the maxima of the 3rd and 4th demodulation harmonics.

We also analyze variations in the probe’s polarizability alongside the near-field increase at the position of the most efficient resonant excitation, where the outer edge fringes reach their maxima. Figure 8a presents vertical scans for hBN at *x*_probe_ = −31 nm, while Figure 8b corresponds to SiC at *x*_probe_ = −37 nm. For the optical material in the hyperbolic regime, the polarizability notably drops as the field enhancement reaches its maximum, in agreement with the resonance model for the planar surface (refer to Figure 3). In contrast, the SiC layer exhibits resonant excitations akin to the LSPh resonance in a sphere, and the polarizability and field enhancement exhibit irregular trends. Nevertheless, for *z*_probe_ > 30 nm, both materials show a decrease in all measured quantities as the probe moves farther from the edge.

The offset between the physical edge position and the signal peak in s-SNOM measurements arises due to a combination of near-field probe interactions, material properties, and edge geometry effects. One key factor is the finite size of the probe apex, which interacts with the sample over a spatially extended region rather than at a single point, leading to a shift in the apparent resonance location. Surface roughness further influences this offset by modifying the local field distribution, effectively broadening or displacing the near-field enhancement region. The impact of surface roughness on s-SNOM response has been extensively studied in both theoretical [75] and experimental works [76,77,78]. As anticipated, higher demodulation harmonics exhibit greater sensitivity to surface protrusions and depressions, enhancing their ability to resolve fine structural details. The analysis has shown that increased roughness can lead to variations in the signal peak position on the order of hundreds of nanometers, depending on the characteristics of the roughness features. For a highly reflective (gold) surface with an 800 nm protrusion, the first harmonic shows an approximate 20% increase in signal, while the third harmonic exhibits a more pronounced enhancement of around 35%, highlighting the amplified response at higher harmonics. Furthermore, the s-SNOM technique has been shown to detect material contrast between gold and silicon beneath a PMMA layer thicker than 100 nm, achieving subsurface resolution below 100 nm [79]. However, a detailed analysis of intricate phenomena, including surface roughness effects and subsurface characterization beyond material contrast detection, lies beyond the scope of this study.

## 5. Comparison with Other Studies and Discussion

Recent studies have introduced new perspectives on s-SNOM by refining signal demodulation techniques, enhancing spatial resolution through higher harmonic analysis, and extending its application to complex nanophotonic and phonon-polaritonic systems [80,81,82,83,84]. Among near-field optical studies, the analysis of edge fringes in phonon-polaritonic materials, such as SiC and hBN, has been relatively scarce. Near-field edge fringes at sharp material boundaries have been investigated in [43], distinguishing between the formation mechanisms of the fringes in both resonant and non-resonant materials. The work has employed full-wave numerical simulations combined with signal demodulation techniques to model the near-field interactions at material edges.

The characteristic features obtained with numerical simulations show good agreement with experimental s-SNOM measurements reported in previous studies for the fringes at the edges of various materials, and the computed resonance positions align well with experimental observations [43,64]. While edge effects have been a subject of study and extensively analyzed theoretically [51], experimental investigations of materials with strong resonant responses and other complex phenomena remain scarce. However, the analysis of edge fringes in phonon-polaritonic materials, such as SiC and hyperbolic hBN, is very rare, with a significant lack of studies systematically investigating their near-field distributions and resonance characteristics. The spatial localization of field enhancements, as well as the demodulated signal variations at different probe heights, closely match measured s-SNOM response maps, confirming the reliability of the theoretical approach. Experimental studies have demonstrated outer bright fringes and strong near-field contrasts at material boundaries, which are accurately reproduced in the simulations, supporting the validity of the effective polarizability model. Moreover, the predicted shifts in resonance features and variations in signal strength with probe position correspond well to experimentally observed trends, reinforcing the applicability of this method for analyzing nanoscale polaritonic phenomena.

The present study advances the field by integrating the probe’s effective polarizability into the simulation framework, enabling a more accurate representation of the tip-sample interaction in s-SNOM. This methodology allows for a more precise prediction of near-field distributions and resonance characteristics at material edges. The simulations reveal that the calculated near-field distributions and resonance positions align well with experimental observations of phonon-polariton excitations in SiC and hBN structures, and this congruence emphasizes the reliability of the theoretical approach employed in this work. However, a limitation of the current method is its partial consideration of higher-order multipolar effects, which may influence the accuracy of the simulated near-field interactions in complex geometries. To further enhance the fidelity of the simulations, future research could focus on fully incorporating these higher-order effects using multipolar decomposition, similar to ones routinely performed for nanoantennas [85,86,87]. Additionally, experimental validation of the simulated edge fringes in various phonon-polaritonic materials will be beneficial for fully establishing the efficacy of the proposed model and investigating the near-field properties of such edges.

While the dipolar approximation used in this work provides valuable insights into the near-field interactions in s-SNOM measurements, it has limitations when applied to nanostructures with very complex geometries and multiple hot spots. In such cases, the probe’s interaction with the sample may involve higher-order multipolar contributions that are not fully captured by the dipolar model, potentially leading to inaccuracies in predicting field distributions and resonance characteristics. A more rigorous approach could involve higher-order multipolar expansions, which account for additional modes of probe scattering and improve the representation of localized field enhancements. Alternatively, full-wave numerical methods, such as the finite element method or finite-difference time-domain method, can be employed to model the complete electromagnetic response of the system without relying on dipolar assumption. Future work could integrate these more advanced techniques to refine the analysis, particularly for structures with strong anisotropic responses or highly confined resonances where multipolar effects play a significant role.

The main challenge lies in extracting the contributions of different multipolar components from the computed near-field interactions and accurately calculating the scattered signal. In the present model, this is achieved by computing the probe’s reflection coefficient under the dipolar approximation, which is then used to estimate the scattered field. However, the rest of the calculations, including the near-field enhancement and demodulation analysis, are not strictly limited to the dipolar assumption. A more comprehensive approach would involve explicitly accounting for higher-order multipolar components in the probe response, allowing for a more accurate reconstruction of the full scattered signal. This requires expanding the model beyond the dipolar approximation and incorporating additional scattering terms that contribute to the experimentally measured s-SNOM response.

The integration of phonon-polariton resonant materials into nanophotonic circuits can enable highly confined and tunable mid-infrared waveguiding, which is crucial for on-chip sensing and spectroscopy applications. By leveraging hyperbolic dispersion in van der Waals materials, such as hexagonal boron nitride, it is possible to design compact optical components, including ultrathin directional couplers and nanoresonators, that surpass conventional diffraction limits. The findings of this study could inform the development of hybrid photonic–electronic platforms where phonon-polaritonic structures facilitate low-loss, subwavelength-scale signal processing for next-generation integrated photonic devices.

## 6. Conclusions

Novel layered materials that exhibit low-loss phonon-polariton resonances hold great potential as components for nanoscale photonic structures, metasurfaces, and functional devices in the mid-IR spectrum. We investigated surface and edge resonance properties through s-SNOM measurement simulations. Resonances on a flat surface or in a nanosphere offer fundamental insights into changes in the probe’s effective polarizability and field enhancement at various locations, which are linked to energy absorption. A key observation in this study is that excitation of resonance results in a reduction in both the effective polarizability and the field at the probe, contrary to the enhanced near fields observed in the nanostructure. To investigate resonant modes in more advanced nanostructures, in particular materials with phonon-polaritons as representative examples, we used the following technique: on the one hand, it involves calculating the s-SNOM response within the dipolar assumption, and on the other hand, it entails demodulating the field enhancement at the probe apex. The s-SNOM response calculations were based on extracting the probe’s effective polarizability from the reflectance, which is heavily influenced by the dipolar approximation. This method is more suited for structures where the probe excites only a single hot spot, such as a planar interface. As the second highlight of the work, we demonstrated that demodulation of field enhancement needs to be used when the probe scattering generates higher-order multipolar components, as in the case of material edges with a thickness similar to the probe apex radius. The proposed method captures the contributions from all hot spots excited near the probe.

The applicability of these two approaches is evaluated for resonant states in nanostructured materials with phonon-polaritons, such as silicon carbide, and this also includes hBN in the mid-IR range in a hyperbolic regime. In materials with hyperbolic dispersion, the resonant modes are associated with the phonon-polaritons as well as their repetitive reflections from the material edges. When the probe coordinates correspond to the peak of the outer bright band and the strongest resonant excitation at the material boundary, the polarizability decreases in the upper ~10s of nanometers near the nanostructure, while the near fields at the boundary are significantly increased. For hBN, both computation techniques yielded profiles that align well. The SiC layer supports resonant states resembling those observed in other nanostructures with phonon-polariton or plasmonic resonances. These resonances were accompanied by complex field distributions and multiple hot spots, leading to multipolar excitations in the probe. In this case, calculating the demodulation of field enhancement is preferred over the computation of the s-SNOM response using polarizability and dipolar assumption.

## Data Availability

The data presented in the author’s plots are available upon request from the author.

## References

[1] Hillenbrand R., Taubner T., Keilmann F. (2002). Phonon-enhanced light–matter interaction at the nanometre scale. Nature.

[2] Dai S., Fei Z., Ma Q., Rodin A.S., Wagner M., McLeod A.S., Liu M.K., Gannett W., Regan W., Watanabe K. (2014). Tunable Phonon Polaritons in Atomically Thin van der Waals Crystals of Boron Nitride. Science.

[3] Low T., Chaves A., Caldwell J.D., Kumar A., Fang N.X., Avouris P., Heinz T.F., Guinea F., Martin-Moreno L., Koppens F. (2017). Polaritons in layered two-dimensional materials. Nat. Mater..

[4] Caldwell J.D., Vurgaftman I., Tischler J.G., Glembocki O.J., Owrutsky J.C., Reinecke T.L. (2016). Atomic-scale photonic hybrids for mid-infrared and terahertz nanophotonics. Nat. Nanotechnol..

[5] Caldwell J.D., Lindsay L., Giannini V., Vurgaftman I., Reinecke T.L., Maier S.A., Glembocki O.J. (2015). Low-loss, infrared and terahertz nanophotonics using surface phonon polaritons. Nanophotonics.

[6] Taubner T., Korobkin D., Urzhumov Y., Shvets G., Hillenbrand R. (2006). Near-Field Microscopy Through a SiC Superlens. Science.

[7] Schuller J.A., Zia R., Taubner T., Brongersma M.L. (2007). Dielectric metamaterials based on electric and magnetic resonances of silicon carbide particles. Phys. Rev. Lett..

[8] Kaps F.G., Kehr S.C., Eng L.M. (2023). Polarization Sensitivity in Scattering-Type Scanning Near-Field Optical Microscopy—Towards Nanoellipsometry. Appl. Sci..

[9] Caldwell J.D., Glembocki O.J., Francescato Y., Sharac N., Giannini V., Bezares F.J., Long J.P., Owrutsky J.C., Vurgaftman I., Tischler J.G. (2013). Low-Loss, Extreme Subdiffraction Photon Confinement via Silicon Carbide Localized Surface Phonon Polariton Resonators. Nano Lett..

[10] Chabi S., Kadel K. (2020). Two-Dimensional Silicon Carbide: Emerging Direct Band Gap Semiconductor. Nanomaterials.

[11] Oglesby S., Ivanov S.A., Londonõ-Calderon A., Pete D., Pettes M.T., Jones A.C., Chabi S. (2022). Manufacturing of Complex Silicon–Carbon Structures: Exploring SixCy Materials. Materials.

[12] Chabi S., Guler Z., Brearley A.J., Benavidez A.D., Luk T.S. (2021). The Creation of True Two-Dimensional Silicon Carbide. Nanomaterials.

[13] Li P., Yang X., Maß T.W.W., Hanss J., Lewin M., Michel A.-K.U., Wuttig M., Taubner T. (2016). Reversible optical switching of highly confined phonon–polaritons with an ultrathin phase-change material. Nat. Mater..

[14] Kumar A., Low T., Fung K.H., Avouris P., Fang N.X. (2015). Tunable Light–Matter Interaction and the Role of Hyperbolicity in Graphene–hBN System. Nano Lett..

[15] Li P., Dolado I., Alfaro-Mozaz F.J., Nikitin A.Y., Casanova F., Hueso L.E., Vélez S., Hillenbrand R. (2017). Optical Nanoimaging of Hyperbolic Surface Polaritons at the Edges of van der Waals Materials. Nano Lett..

[16] Ahmed H., Babicheva V.E. (2020). Nanostructured Tungsten Disulfide WS2 as Mie Scatterers and Nanoantennas. MRS Adv..

[17] Sun J., Litchinitser N.M., Zhou J. (2014). Indefinite by Nature: From Ultraviolet to Terahertz. ACS Photonics.

[18] Ambrosio A., Jauregui L.A., Dai S., Chaudhary K., Tamagnone M., Fogler M.M., Basov D.N., Capasso F., Kim P., Wilson W.L. (2017). Mechanical Detection and Imaging of Hyperbolic Phonon Polaritons in Hexagonal Boron Nitride. ACS Nano.

[19] Poddubny A., Iorsh I., Belov P., Kivshar Y. (2013). Hyperbolic metamaterials. Nat. Photonics.

[20] Chebykin A.V., Babicheva V.E., Iorsh I.V., Orlov A.A., Belov P.A., Zhukovsky S.V. (2016). Enhancement of the Purcell factor in multiperiodic hyperboliclike metamaterials. Phys. Rev. A.

[21] Ishii S., Kildishev A.V., Narimanov E., Shalaev V.M., Drachev V.P. (2013). Sub-wavelength interference pattern from volume plasmon polaritons in a hyperbolic medium. Laser Photonics Rev..

[22] Babicheva V.E. (2024). Resonant Metasurfaces with Van Der Waals Hyperbolic Nanoantennas and Extreme Light Confinement. Nanomaterials.

[23] Babicheva V.E. (2018). Lattice Kerker effect in the array of hexagonal boron nitride antennas. MRS Adv..

[24] Babicheva V.E. (2018). Directional scattering by the hyperbolic-medium antennas and silicon particles. MRS Adv..

[25] Alfaro-Mozaz F.J., Alonso-González P., Vélez S., Dolado I., Autore M., Mastel S., Casanova F., Hueso L.E., Li P., Nikitin A.Y. (2017). Nanoimaging of resonating hyperbolic polaritons in linear boron nitride antennas. Nat. Commun..

[26] Li P., Lewin M., Kretinin A.V., Caldwell J.D., Novoselov K.S., Taniguchi T., Watanabe K., Gaussmann F., Taubner T. (2015). Hyperbolic phonon-polaritons in boron nitride for near-field optical imaging and focusing. Nat. Commun..

[27] Dai S., Ma Q., Andersen T., Mcleod A.S., Fei Z., Liu M.K., Wagner M., Watanabe K., Taniguchi T., Thiemens M. (2015). Subdiffractional focusing and guiding of polaritonic rays in a natural hyperbolic material. Nat. Commun..

[28] E Babicheva V. (2017). Long-range propagation of plasmon and phonon polaritons in hyperbolic-metamaterial waveguides. J. Opt..

[29] Nikitin A.Y., Yoxall E., Schnell M., Vélez S., Dolado I., Alonso-Gonzalez P., Casanova F., Hueso L.E., Hillenbrand R. (2016). Nanofocusing of Hyperbolic Phonon Polaritons in a Tapered Boron Nitride Slab. ACS Photonics.

[30] Chen X., Hu D., Mescall R., You G., Basov D.N., Dai Q., Liu M. (2019). Modern Scattering-Type Scanning Near-Field Optical Microscopy for Advanced Material Research. Adv. Mater..

[31] Wang C.-F., Kafle B., Tesema T.E., Kookhaee H., Habteyes T.G. (2020). Molecular Sensitivity of Near-Field Vibrational Infrared Imaging. J. Phys. Chem. C.

[32] Raschke M.B., Schubert M., Narang P., Paarmann A. (2023). Optical nanoprobe imaging and spectroscopy. Appl. Phys. Lett..

[33] O’callahan B.T., Jones A.C., Park J.H., Cobden D.H., Atkin J.M., Raschke M.B. (2015). Inhomogeneity of the ultrafast insulator-to-metal transition dynamics of VO_2_. Nat. Commun..

[34] Atkin J.M., Berweger S., Jones A.C., Raschke M.B. (2012). Nano-Optical Imaging and Spectroscopy of Order, Phases, and Domains in Complex Solids. Adv. Phys..

[35] Moreno C., Alda J., Kinzel E., Boreman G. (2017). Phase imaging and detection in pseudo-heterodyne scattering scanning near-field optical microscopy measurements. Appl. Opt..

[36] Zhao Z., Kravtsov V., Wang Z., Zhou Z., Dou L., Huang D., Wang Z., Cheng X., Raschke M.B., Jiang T. (2025). Applications of ultrafast nano-spectroscopy and nano-imaging with tip-based microscopy. eLight.

[37] Nan C., Yue W., Tao L., Yang X. (2020). Fourier transform infrared nano-spectroscopy: Mechanism and applications. Appl. Spectrosc. Rev..

[38] Chen X., Xu S., Shabani S., Zhao Y., Fu M., Millis A.J., Fogler M.M., Pasupathy A.N., Liu M., Basov D.N. (2023). Machine Learning for Optical Scanning Probe Nanoscopy. Adv. Mater..

[39] Kwon S., Kim J.M., Ma P.J., Guan W., Nam S. (2023). Near-Field Nano-Optical Imaging of van der Waals Materials. Adv. Phys. Res..

[40] Hauer B., Marvinney C.E., Lewin M., Mahadik N.A., Hite J.K., Bassim N., Giles A.J., Stahlbush R.E., Caldwell J.D., Taubner T. (2020). Exploiting Phonon-Resonant Near-Field Interaction for the Nanoscale Investigation of Extended Defects. Adv. Funct. Mater..

[41] Stiegler J.M., Abate Y., Cvitkovic A., Romanyuk Y.E., Huber A.J., Leone S.R., Hillenbrand R. (2011). Nanoscale Infrared Absorption Spectroscopy of Individual Nanoparticles Enabled by Scattering-Type Near-Field Microscopy. ACS Nano.

[42] Govyadinov A.A., Mastel S., Golmar F., Chuvilin A., Carney P.S., Hillenbrand R. (2014). Recovery of Permittivity and Depth from Near-Field Data as a Step toward Infrared Nanotomography. ACS Nano.

[43] Babicheva V.E., Gamage S., Stockman M.I., Abate Y. (2017). Near-field edge fringes at sharp material boundaries. Opt. Express.

[44] Cvitkovic A., Ocelic N., Hillenbrand R. (2007). Material-Specific Infrared Recognition of Single Sub-10 nm Particles by Substrate-Enhanced Scattering-Type Near-Field Microscopy. Nano Lett..

[45] Babicheva V.E., Gamage S., Zhen L., Cronin S.B., Yakovlev V.S., Abate Y. (2018). Near-field Surface Waves in Few-Layer MoS2. ACS Photonics.

[46] Woessner A., Lundeberg M.B., Gao Y., Principi A., Alonso-González P., Carrega M., Watanabe K., Taniguchi T., Vignale G., Polini M. (2015). Highly confined low-loss plasmons in graphene–boron nitride heterostructures. Nat. Mater..

[47] Dai S., Ma Q., Liu M.K., Andersen T., Fei Z., Goldflam M.D., Wagner M., Watanabe K., Taniguchi T., Thiemens M. (2015). Graphene on hexagonal boron nitride as a tunable hyperbolic metamaterial. Nat. Nanotechnol..

[48] Habteyes T.G., Staude I., Chong K.E., Dominguez J., Decker M., Miroshnichenko A., Kivshar Y., Brener I. (2014). Near-Field Mapping of Optical Modes on All-Dielectric Silicon Nanodisks. ACS Photonics.

[49] McLeod A.S., Kelly P., Goldflam M.D., Gainsforth Z., Westphal A.J., Dominguez G., Thiemens M.H., Fogler M.M., Basov D.N. (2014). Model for quantitative tip-enhanced spectroscopy and the extraction of nanoscale-resolved optical constants. Phys. Rev. B.

[50] Lu G., Zhao R., Yin H., Xiao Z., Zhang J. (2020). Improved Point Dipole Model for Subwavelength Resolution Scattering Near-Field Optical Microscopy (SNOM). Int. J. Antennas Propag..

[51] Chen X., Yao Z., Stanciu S.G., Basov D., Hillenbrand R., Liu M. (2021). Rapid simulations of hyperspectral near-field images of three-dimensional heterogeneous surfaces. Opt. Express.

[52] Mohun D., Sulollari N., Salih M., Li L.H., Cunningham J.E., Linfield E.H., Davies A.G., Dean P. (2024). Terahertz microscopy using laser feedback interferometry based on a generalised phase-stepping algorithm. Sci. Rep..

[53] Zhang L.M., Andreev G.O., Fei Z., McLeod A.S., Dominguez G., Thiemens M., Castro-Neto A.H., Basov D.N., Fogler M.M. (2012). Near-field spectroscopy of silicon dioxide thin films. Phys. Rev. B.

[54] Neuman T., Alonso-González P., Garcia-Etxarri A., Schnell M., Hillenbrand R., Aizpurua J. (2015). Mapping the near fields of plasmonic nanoantennas by scattering-type scanning near-field optical microscopy. Laser Photonics Rev..

[55] Maissen C., Chen S., Nikulina E., Govyadinov A., Hillenbrand R. (2019). Probes for Ultrasensitive THz Nanoscopy. ACS Photonics.

[56] Mooshammer F., Huber M.A., Sandner F., Plankl M., Zizlsperger M., Huber R. (2020). Quantifying Nanoscale Electromagnetic Fields in Near-Field Microscopy by Fourier Demodulation Analysis. ACS Photonics.

[57] Wang Y., Wei Y., Zhang Z., Xu X., Bin Z., Zhang T., Zhang X., Liu S., Hu M. (2025). Numerical temporal-spectral analysis of non-scattering THz nanoscopy with resonant cantilevered tips. Opt. Express.

[58] Conrad G., Casper C., Ritchie E.T., Atkin J., Conrad W. (2022). Quantitative modeling of near-field interactions incorporating polaritonic and electrostatic effects. Opt. Express.

[59] McArdle P., Lahneman D.J., Biswas A., Keilmann F., Qazilbash M.M. (2020). Near-field infrared nanospectroscopy of surface phonon-polariton resonances. Phys. Rev. Res..

[60] Datz D., Németh G., Rátkai L., Pekker Á., Kamarás K. (2023). Generalized Mie Theory for Full-Wave Numerical Calculations of Scattering Near-Field Optical Microscopy with Arbitrary Geometries. Phys. Status Solidi (RRL)–Rapid Res. Lett..

[61] Mester L., Govyadinov A.A., Hillenbrand R. (2022). High-fidelity nano-FTIR spectroscopy by on-pixel normalization of signal harmonics. Nanophotonics.

[62] Jiang B.-Y., Zhang L.M., Neto A.H.C., Basov D.N., Fogler M.M. (2016). Generalized spectral method for near-field optical microscopy. J. Appl. Phys..

[63] Wang L., Xu X.G. (2015). Scattering-type scanning near-field optical microscopy with reconstruction of vertical interaction. Nat. Commun..

[64] Abate Y., Gamage S., Li Z., Babicheva V., Javani M.H., Wang H., Cronin S.B., Stockman M.I. (2016). Nanoscopy Reveals Metallic Black Phosphorus. Light Sci. Appl..

[65] Rakić A.D., Djurišić A.B., Elazar J.M., Majewski M.L. (1998). Optical properties of metallic films for vertical-cavity optoelectronic devices. Appl. Opt..

[66] Babar S., Weaver J.H. (2015). Optical constants of Cu, Ag, and Au revisited. Appl. Opt..

[67] Taubner T., Hillenbrand R., Keilmann F. (2003). Performance of visible and mid-infrared scattering-type near-field optical microscopes. J. Microsc..

[68] Taubner T., Keilmann F., Hillenbrand R., Eilmann F. (2005). Nanoscale-resolved subsurface imaging by scattering-type near-field optical microscopy. Opt. Express.

[69] Raschke M.B., Lienau C. (2003). Apertureless near-field optical microscopy: Tip–sample coupling in elastic light scattering. Appl. Phys. Lett..

[70] Mastel S., Govyadinov A.A., Maissen C., Chuvilin A., Berger A., Hillenbrand R. (2018). Understanding the Image Contrast of Material Boundaries in IR Nanoscopy Reaching 5 nm Spatial Resolution. ACS Photonics.

[71] Chen X., Yao Z., Sun Z., Stanciu S.G., Basov D., Hillenbrand R., Liu M. (2022). Rapid simulations of hyperspectral near-field images of three-dimensional heterogeneous surfaces—Part II. Opt. Express.

[72] Kazantsev D., Ryssel H. (2020). ASNOM mapping of SiC epilayer doping profile and of surface phonon polariton waveguiding. J. Appl. Phys..

[73] Cai Y., Zhang L., Zeng Q., Cheng L., Xu Y. (2007). Infrared reflectance spectrum of BN calculated from first principles. Solid State Commun..

[74] Nemilentsau A., Low T., Hanson G. (2016). Anisotropic 2D Materials for Tunable Hyperbolic Plasmonics. Phys. Rev. Lett..

[75] Ge S., Zhang D., Peng Z., Meng J., Ge Q., Peng M. (2023). Rough surface effect in terahertz near-field microscopy: 3D simulation analysis. Appl. Opt..

[76] Ge S., Zhang D., Peng Z., Wang L., Li H. Nano-Scale Rough Surface Imaging with THz-s-SNOM. Proceedings of the 2024 15th Global Symposium on Millimeter-Waves & Terahertz (GSMM).

[77] Yakubovsky D.I., Grudinin D.V., Ermolaev G.A., Vyshnevyy A.A., Mironov M.S., Novikov S.M., Arsenin A.V., Volkov V.S. (2023). Scanning Near-Field Optical Microscopy of Ultrathin Gold Films. Nanomaterials.

[78] Abate Y., Babicheva V.E., Yakovlev V., Dietz N., Feng Z.C. (2017). Chapter 6: Towards understanding and control of nanoscale phase segregation in indium-gallium-nitride alloys. III-Nitride Materials, Devices, and Nano-Structures.

[79] Zhang W., Chen Y. (2020). Visibility of subsurface nanostructures in scattering-type scanning near-field optical microscopy imaging. Opt. Express.

[80] Hillenbrand R., Abate Y., Liu M., Chen X., Basov D.N. (2025). Visible-to-THz near-field nanoscopy. Nat. Rev. Mater..

[81] Kwon S., Ma P.J., Lum C., Hajarian A., Seo J., Nam S. (2025). Nano-optical metrologies for characterizing the carrier dynamics in two-dimensional materials. Mater. Res. Bull..

[82] Chen S., Bylinkin A., Wang Z., Schnell M., Chandan G., Li P., Nikitin A.Y., Law S., Hillenbrand R. (2022). Real-space nanoimaging of THz polaritons in the topological insulator Bi_2_Se_3_. Nat. Commun..

[83] Wang H., Li J., Edgar J.H., Xu X.G. (2020). Three-dimensional near-field analysis through peak force scattering-type near-field optical microscopy. Nanoscale.

[84] Guo X., Bertling K., Rakić A.D. (2021). Optical constants from scattering-type scanning near-field optical microscope. Appl. Phys. Lett..

[85] Han A., Moloney J.V., Babicheva V.E. (2022). Applicability of multipole decomposition to plasmonic- and dielectric-lattice resonances. J. Chem. Phys..

[86] Romero A., Babicheva V.E. (2024). Enhanced light confinement in nonlocal resonant metasurfaces with weak multipolar scatterers. J. Appl. Phys..

[87] Babicheva V.E., Rumi M. (2024). Chalcophosphate metasurfaces with multipolar resonances and electro-optic tuning. RSC Adv..

